# Long Intergenic Non-protein Coding RNA 511 in Cancers

**DOI:** 10.3389/fgene.2020.00667

**Published:** 2020-07-07

**Authors:** Xiao-Fei Wang, Bo Liang, Cheng Chen, Da-Xiong Zeng, Yu-Xiu Zhao, Nan Su, Wei-Wei Ning, Wen Yang, Jian-An Huang, Ning Gu, Ye-Han Zhu

**Affiliations:** ^1^Department of Pulmonary and Critical Care Medicine, The First Affiliated Hospital of Soochow University, Suzhou, China; ^2^Nanjing University of Chinese Medicine, Nanjing, China; ^3^Hospital of Traditional Chinese Medicine (T.C.M) Affiliated to Southwest Medical University, Luzhou, China; ^4^Nanjing Hospital of Chinese Medicine Affiliated to Nanjing University of Chinese Medicine, Nanjing, China

**Keywords:** LINC00511, prognostic biomarker, survival, meta-analysis, bioinformatics, cancer

## Abstract

**Background:** Long intergenic non-protein coding RNA 511 (LINC00511) is upregulated in diverse cancers and involved in prognosis. This study aimed to evaluate the prognostic profile of LINC00511 in cancer patients.

**Methods:** Published studies evaluating the prognosis of LINC00511 in patients with different cancers were identified from Medline, Embase, and Web of Science. Analysis of the association between LINC00511 and clinicopathological characteristics was conducted. GEPIA was used to validation and functional analysis and LnCeVar was used to get genomic variations.

**Results:** We eventually included 9 studies, and the combined results showed LINC00511 was significantly associated with decreased OS (HR = 3.18, 95% CI = 2.29 ~ 4.42, *P* < 0.001) albeit with mild heterogeneity (*I*^2^ = 58.1%, *P*_h_ = 0.014), similarly in cancer type subgroups: breast cancer, digestive system cancer, and cervical cancer (all *P* < 0.001). There is no publication bias and meta-regression indicated follow-up time maybe heterogeneity of the results (*P* = 0.008). Additionally, LINC00511 appeared to be correlated with age, clinical stage, tumor size, and lymph node metastasis. Those findings were confirmed in GEPIA. Through LnCeVars, gene ontology and functional pathways were enriched, and dysregulated hallmarks and related ceRNA network of LINC00511 were disturbed.

**Conclusions:** LINC00511 could be predictive of poor OS and lymph node metastasis in multiple cancers, in another word, LINC00511 serves as an unfavorable prognostic factor, and its mechanism is related to ceRNA.

## Introduction

Cancer is becoming a major threat to human health and a major public health problem worldwide with its increasing morbidity and mortality (Chen et al., 2016). However, according to the degree of economic development and social and lifestyle factors, the most commonly diagnosed cancers and major cancer deaths vary greatly between and within countries. It is estimated by the International Agency for Research on Cancer (IARC) that there are 18.1 million new cancer cases and 9.6 million cancer deaths worldwide in 2018 (Bray et al., 2018; Ferlay et al., 2019). Although there has been some alleviation in cancer incidence and mortality in recent decades, such as in the United States, the cancer incidence rate from 2006 to 2015 declined by around 2% per year in men and was stable in women, and the cancer death rate from 2007 to 2016 declined annually by only 1.5% in both men and women (Siegel et al., 2019), it is still a problem that we urgently need to address. Reducing the risk factors relating cancers is effective in preventing many estimated cases and deaths (Chen et al., 2016; Siegel et al., 2019), but this is clearly not enough, we need early detection, diagnosis, and treatment.

As the improved technology of RNA-seq is being widely applied, there is a deeper appreciation for the transcriptome of organisms. Long non-coding RNAs (lncRNAs) are a kind of different RNAs, which has longer than 200 nt transcripts with disability of encoding proteins (Zhang X. et al., 2019). In-depth studies on lncRNAs can not only explain biological physiological and pathological processes, but also provide novel perspectives for the diagnosis, prevention and treatment of clinical diseases (Zhang X. et al., 2019), especially malignant tumors (Bhan et al., 2017), as it involves abundant biological processes throughout life (Quinn and Chang, 2016). LncRNAs has also been considered as a biomarker for diagnosis and prognosis, which may provide a target for potential therapeutic applications (Bhan et al., 2017). However, their potential mechanisms have yet to be confirmed. It is more reliable to solve the potential application of lncRNAs in prognosis of cancers (Zhou et al., 2019), therefore, than to develop targeted therapies (Liang et al., 2018). RNA-seq of total RNA from 20 human tissues showed that long intergenic non-protein coding RNA 511 (LINC00511) is highly expressed in the small intestine, stomach, salivary gland and lung (Duff et al., 2015). Moreover, numerous evidence have demonstrated that LINC00511 plays a vital role on various cancer progression and tumorigenesis, like hepatocellular carcinoma (Wang R. P. et al., 2019), bladder cancer (Li et al., 2018), breast cancer (Lu et al., 2018; Liu et al., 2019; Zhang J. et al., 2019), cervical cancer (Mao et al., 2019; Yu et al., 2019), clear cell renal cell carcinoma (Deng et al., 2019), non-small cell lung cancer (NSCLC) (Sun et al., 2016), pancreatic ductal adenocarcinoma (Zhao et al., 2018), tongue squamous cell carcinoma (Ding et al., 2018) and pan-cancer (Cabanski et al., 2015). LINC00511 has various functions, including cell development, regulation of developmental process, apoptosis, and programmed cell death, among hemostasis, various cancers, and focal adhesion, based on molecular pathways and biological processes (Wang et al., 2019a).

A broader application of existing knowledge would undoubtedly accelerate progress against cancer. We also hope to make a contribution here. Accordingly, we reviewed the pertinent literatures and searched GEPIA (Tang et al., 2017) to conduct the current study to explore the clinicopathological and prognostic value of LINC00511 and discussed their potential implications and mechanisms in different types of cancer through LnCeVars (Wang et al., 2019a).

## Methods

### Search Strategy and Selection Criteria

Two reviewers (XFW and BL) searched Medline, Embase and Web of Science for cohort studies comparing the relationship between LINC00511 expression and cancers before October 20, 2019, with language limited to English. The terms searched were: neoplasia, tumor, cancer, malignancy, carcinoma, LINC00511, Long Intergenic Non-Protein Coding RNA 511. Details of the search strategy are reported in Table S1.

Inclusion criteria for studies: (1) participants were pathologically confirmed with cancer; (2) the expression of LINC00511 in tissues or plasma was detected; (3) patients with LINC00511 high and low expression group; (4) the relationship between LINC00511 and tumor prognosis [such as overall survival (OS), progression-free survival (PFS), disease-free survival (DFS) and disease-specific survival (DSS)] or clinicopathological features was described; (5) relevant data was sufficient to obtain hazard ratio (HR) and 95% confidence interval (CI).

Exclusion criteria included reviews, case reports, comments, expert opinions, meta-analysis, and conference summaries. When studies or data duplication were found, only the latest or most complete studies were included.

### Data Extraction and Management

Two reviewers (CC and DXZ) extracted the information about study characteristics, demographic characteristics, the expression of LINC00511 as well as outcomes from remaining studies. If univariate analysis and multivariate analysis are both included in the study, priority is given to extract multivariate analysis result. For studies that did not provide HRs and 95% CIs directly, we estimated them through indirect methods (Tierney et al., 2007). If there were exact HRs, except Kaplan-Meier curves, HRs were obtained from those curves though Engauge Digitizer (Parmer et al., 1998). Then the third reviewer (YXZ) checked received data. If there were any contradictory, it was decided by the final reviewer (NS). Finally, all extracted data were stored in the predesigned excel spreadsheet.

### Quality Assessment

Two authors (XFW and BL) applied Newcastle Ottawa Scale (NOS), including selection, comparability and outcome, to assessed selected studies critically and independently (Stang, 2010; Chu-Ling et al., 2016). The studies were divided into three grades: low, medium, and high, with NOS scores ranging from 0 to 3, 4 to 6, and 7 to 9, respectively. The third reviewer (YXZ) decided in contradictory cases, and the results were also shown in the predesigned excel spreadsheet.

### Data Synthesis and Analysis

The STATA 12.0 (Stata Corp., College Station, TX, USA), a statistical software for data science, was adopted to synthesis and analysis data. The fixed-effect model was chosen to synthesis highly homogeneous data, whereas, the random-effect model and subgroup analysis were conducted for heterogeneous data. Moreover, sensitivity analysis was conducted to assess heterogeneity. We would assign adjectives of low, moderate, and high to *I*^2^ values of 25, 50, and 75% as described previously (Higgins et al., 2003; Liang et al., 2019; Wang et al., 2020). Lastly, the funnel plot and Egger's tests were implemented to assess the publication bias, and meta-regression analysis was applied to trace the origin of heterogeneity.

### Bioinformatics Analysis

GEPIA was performed to obtain the information of LINC00511 across cancer and adjacent normal tissues and relevant prognostic information. Then the expression of LINC00511 among several cancers was plotted and OS and DFS were evaluated. LnCeVars, a comprehensive database synthesizing published studies and abundant datasets (Wang et al., 2019a), was used to get competing endogenous RNA (ceRNA) network regulation.

## Results

### Characteristics of Eligible Studies

**Figure 1:**
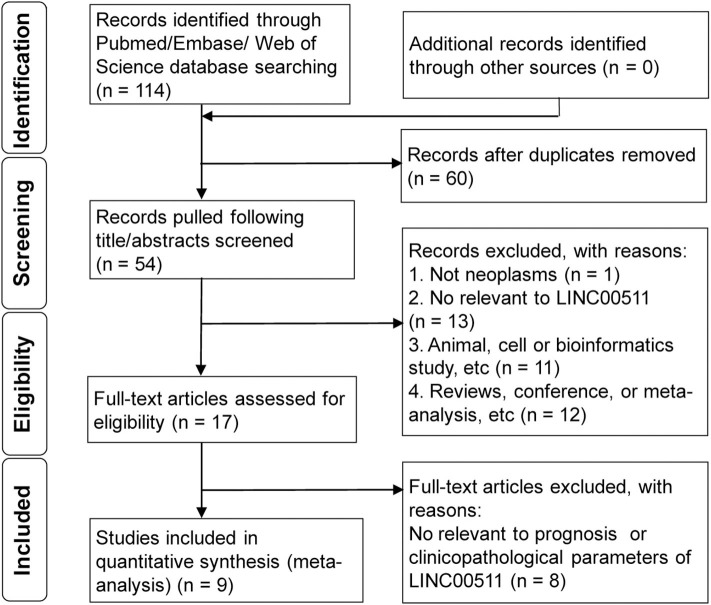
The flow diagram of this meta-analysis.

The initial search of all sources yielded 114 articles eligible for identification. Fifty-fourthtitle/abstracts were screened for eligibility after removing 60 duplicates. Of these, 17 full-text articles were assessed for eligibility. Finally, a total of 9 studies (Sun et al., 2016; Lu et al., 2018; Zhao et al., 2018; Deng et al., 2019; Liu et al., 2019; Mao et al., 2019; Wang R. P. et al., 2019; Yu et al., 2019; Zhang J. et al., 2019) were included in this study (Figure 1).

Nine studies comprising 823 patients contained OS information. All included studies were published in 2016 and later, and all the studies were conducted in China involving NSCLC (Sun et al., 2016), breast cancer (Lu et al., 2018; Liu et al., 2019; Zhang J. et al., 2019), pancreatic ductal adenocarcinoma (Zhao et al., 2018), clear cell renal cell carcinoma (Deng et al., 2019), cervical cancer (Mao et al., 2019; Yu et al., 2019), and hepatocellular carcinoma (Wang R. P. et al., 2019). Only two studies did not report TNM stage (Zhao et al., 2018; Liu et al., 2019). LINC00511 expression was tested by RT-PCR and most of the follow-up duration were more than 5 years. Some HRs were extracted directly from corresponding studies (Sun et al., 2016; Zhao et al., 2018; Yu et al., 2019), and some were extracted from corresponding curves (Lu et al., 2018; Deng et al., 2019; Liu et al., 2019; Mao et al., 2019; Wang R. P. et al., 2019; Zhang J. et al., 2019). The included studies also had different definitions of cut-off value. NOS scores of all included studies were <7. Main characteristics of eligible studies were shown in Table 1.

**Table 1:** The main characteristics of the included studies in this meta-analysis.

	**Country**	**Type**	**NO. (M/F)**	**TNM Stage**	**Method**	**Cut-off**	**High (%)**	**Follow-up**	**Outcome**	**HR estimation**	**NOS**
Sun et al. (2016)	China	NSCLC	124 (58/66)	I24/II-IV100	qRT-PCR	≥2.23-fold	93 (75%)	42 m	OS, CP	OS R (MA)	8
Lu et al. (2018)	China	BC	39 (0/39)	I-II13/III-IV26	qRT-PCR	Mean	23 (59%)	60 m	OS, CP	OS Curve	8
Zhao et al. (2018)	China	PDA	140 (78/62)	NR	qRT-PCR	log2 FC (cancer/normal) > 1	102 (72.9%)	60 m	OS, CP	OS R (MA)	7
Deng et al. (2019)	China	ccRCC	49 (21/28)	I-II26/III-IV23	qRT-PCR	Median	25 (51%)	50 m	OS, CP	OS Curve	8
Liu et al. (2019)	China	BC	98 (0/98)	NR	qRT-PCR	Median	49 (50%)	60 m	OS, CP	OS Curve	9
Mao et al. (2019)	China	CC	84 (0/84)	IIa 29/IIb-II2b 55	qRT-PCR	Median	49 (58.3%)	60 m	OS, CP	OS Curve	9
Wang R. P. et al. (2019)	China	HC	127 (76/51)	I-II79/III-IV49	qRT-PCR	Median	64 (50.4%)	60 m	OS, CP	OS Curve	7
Yu et al. (2019)	China	CC	92 (0/92)	I-IIa38/IIb-IV54	qRT-PCR	Median	46 (50%)	108 m	OS, CP	OS R (MA)	7
Zhang J. et al. (2019)	China	BC	70 (0/70)	I-II65/III-IV5	qRT-PCR	NR	24 (34.3%)	2300 d	OS, CP	OS Curve	7

*BC, breast cancer; CC, cervical cancer; ccRCC, clear cell renal cell carcinoma; CP, clinical parameter; d, days; F, female; FC, fold changes; HC, hepatocellular carcinoma; HR, hazard ratio; M, male; m, months; MA, multivariate analysis; NO, number; NOS, Newcastle Ottawa scale quality criteria.; NR, no report; NSCLC, non-small cell lung cancer; OS, overall survival; PDA, pancreatic ductal adenocarcinoma; qRT-PCR, quantitative real-time polymerase chain reaction; R, reported in text*.

### Association Between LINC00511 and Cancers

**Figure 2:**
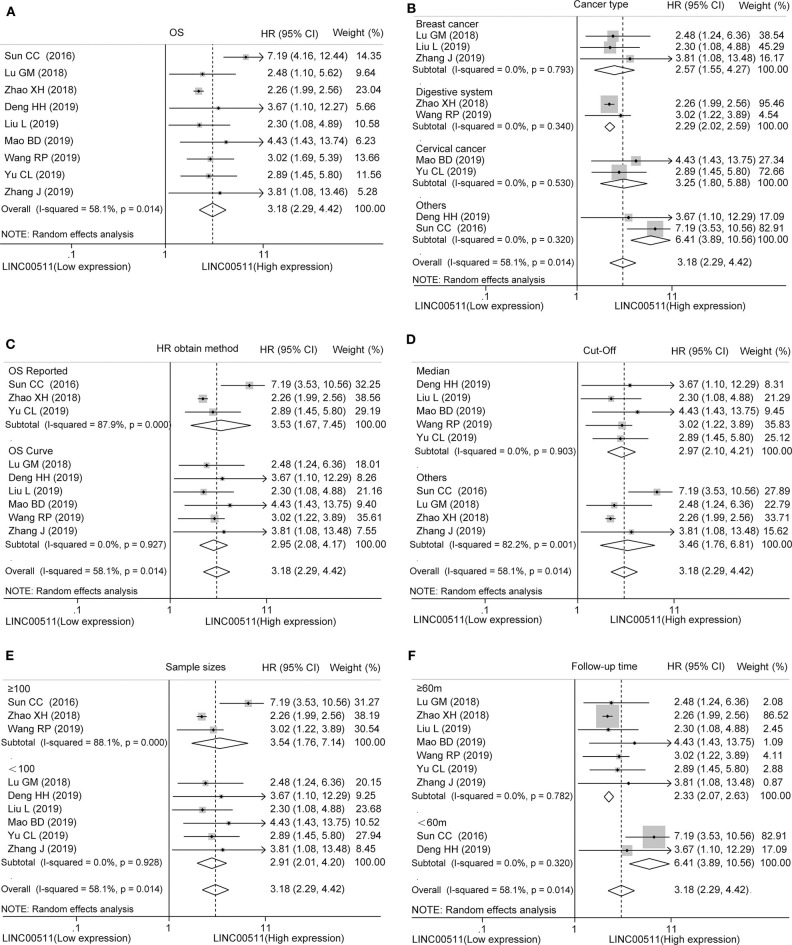
Forest plots of hazard ratios for the association between LINC00511 expression and overall survival. **(A)** Overall survival, subgroup analysis based on **(B)** different cancer types, **(C)** HR obtain method, **(D)** cut-off, **(E)** sample size and **(F)** follow up time.

We analyzed the difference in LINC00511 expression in included studies. As a result, we found that upregulated LINC00511 expression was associated with poor OS in various cancers (HR = 3.18, 95% CI = 2.29 ~ 4.42, *P* < 0.001), albeit with mild heterogeneity (*I*^2^ = 58.1%, *P*_h_ = 0.014) (Figure 2A, Table S2).

### Heterogeneity and Publication Bias

**Figure 3:**
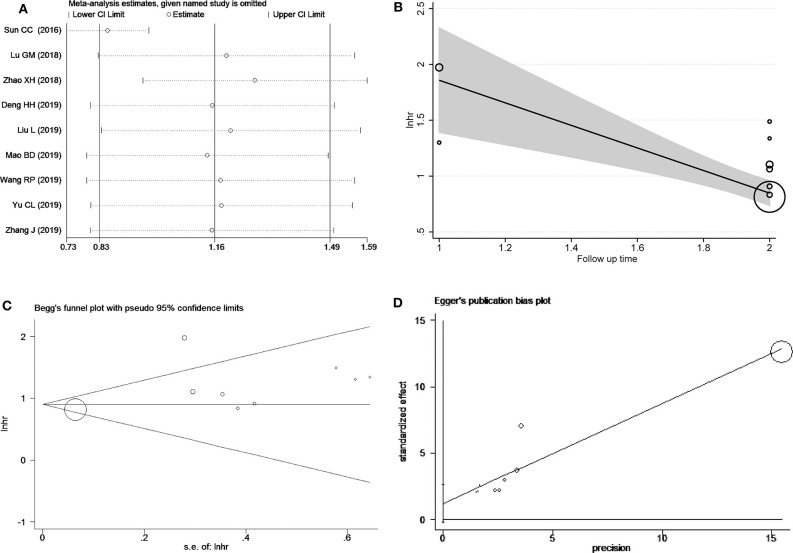
Heterogeneity analysis for the meta-analysis of OS including sensitivity analysis **(A)**, Bgger's test **(B)**, Egger's test **(C)** and meta-regression **(D)**.

Sensitivity analysis of OS was performed to evaluate individual study's influence on the pooled results to verify the consistency of the meta-analysis consequences. The results disclosed Sun et al. study (Sun et al., 2016) may be the source of heterogeneity (Figure 3A). When the study by Sun et al. was omitted, the pooled results changed a lot (HR = 2.34, 95% CI = 2.08 ~ 2.64) and without heterogeneity (*I*^2^ = 0.0%, *P*_h_ = 0.808) (Figure S1). Though meta-regression, we found that follow-up time occupied 96.73% heterogeneity of the results (*P* = 0.008) (Figure 3B). The Begg's funnel plot indicated no publication bias analysis of overall survival (*P* = 0.754) (Figure 3C). Moreover, Egger's test was applied to confirmed (*P* = 0.082) (Figure 3D).

### Subgroup Analysis

Furthermore, in order to understand the impact of various factors on OS, we conducted a subgroup analysis stratified by cancer types, HR obtain method, cut-off, sample size and follow up time. The results revealed that LINC00511 could act as prognostic indicator of OS for patients with breast cancer (HR = 2.57, 95% CI = 1.55 ~ 4.27, *P* < 0.001), digestive system cancer (HR = 2.29, 95% CI = 2.02 ~ 2.59, *P* < 0.001), and cervical cancer (HR = 3.25, 95% CI = 1.80 ~ 5.88, *P* < 0.001) (Figure 2B, Table S2). After stratification by HR obtain method, we found that LINC00511 was a prognostic factor whether HR was obtained directly (HR = 3.53, 95% CI = 1.67 ~ 7.45, *P* = 0.001) or indirectly (HR = 2.95, 95% CI = 2.08 ~ 4.17, *P* < 0.001) (Figure 2C, Table S2). We also conducted subgroup analysis on whether the median expression of LINC00511 was used as the cut-off value to divide the high expression and low expression, and the results showed that higher LINC00511 was associated with poor prognosis, regardless the cut-off value (Figure 2D, Table S2). Subsequently, we found that LINC00511 could act as a prognostic factor in groups with sample size >= 100 patients (HR = 3.54, 95% CI = 1.76 ~ 7.14, *P* < 0.001) or groups with sample size <100 patients (HR = 2.91, 95% CI = 2.01 ~ 4.20, *P* < 0.001) (Figure 2E, Table S2). Subgroup analysis of the follow-up time showed that up-expression of LINC00511 related to poor OS in long follow-up time (>= 60 months, HR = 2.34, 95% CI = 2.08 ~ 2.63, *P* < 0.001) and, to a larger extent, in short follow-up time (<60 months, HR = 6.41, 95% CI = 3.89 ~ 10.56, *P* < 0.001) (Figure 2F, Table S2).

### Association Between LINC00511 and Clinicopathological Characteristics

Not all of the included studies discussed the relationship between LINC00511 and clinicopathological characteristics of cancers. Except the literatures about breast cancer and cervical cancer, 4 studies showed there was no relationship between LINC00511 and gender (*P* = 0.27) (Table 2, Figure S2). The same results were found in differentiation and metastasis (*P* = 0.156, 0.435, respectively) (Table 2, Figures S2E,G). The old NSCLC patients had a lower LINC00511 expression than young patients (OR = 0.67, 95% CI = 0.50 ~ 0.90, *P* = 0.008) (Table 2, Figure S2B). The patients with clinical stage III ~ IV exposed LINC00511 up-expression compared with those with clinical stage I ~ II cancer (OR = 2.55, 95% CI = 1.47 ~ 4.42, *P* = 0.001) (Table 2, Figure S2C). Patients with large tumor size had elevated LINC00511 expression than those with small tumor size (OR = 3.29, 95% CI = 1.81 ~ 5.98, *P* < 0.001) (Table 2, Figure S2D). Moreover, cancer patients with lymph node metastasis displayed LINC00511 overexpression than those without lymph node metastasis (OR = 3.03, 95% CI = 1.77 ~ 5.20, *P* < 0.001) (Table 2, Figure S2F). There was no obvious asymmetry in funnel plots of clinical parameters (Figure S3).

**Table 2:** Meta-analysis of the association between LINC00511 expression and clinicopathological characteristics.

**Clinicopathological parameter**	**Number of studies**	**Odds ratio (95% CI)**	***P*-value**	***I*^**2**^ (%)**	**Model**
Gender (male vs. female)	4	0.80 (0.53 ~ 1.19)	0.27	0.00%	Fixed
Age (old vs. young)	9	0.67 (0.50 ~ 0.90)	**0.008**	0.00%	Fixed
Clinical stage (III–IV vs. I–II)	4	2.55 (1.47 ~ 4.42)	**0.001**	0.00%	Fixed
Tumor size (large vs. small)	8	3.29 (1.81 ~ 5.98)	**<0.001**	62.30%	Random
Differentiation (poor vs. good)	5	1.33 (0.90 ~ 1.96)	0.156	0.00%	Fixed
Lymph node metastasis (yes vs. no)	8	3.03 (1.77 ~ 5.20)	**<0.001**	52.40%	Random
Metastasis (yes vs. no)	4	1.47 (0.56 ~ 3.83)	0.435	59.70%	Random

*Bold values indicate <0.05*.

### Validation in GEPIA Dataset

**Figure 4:**
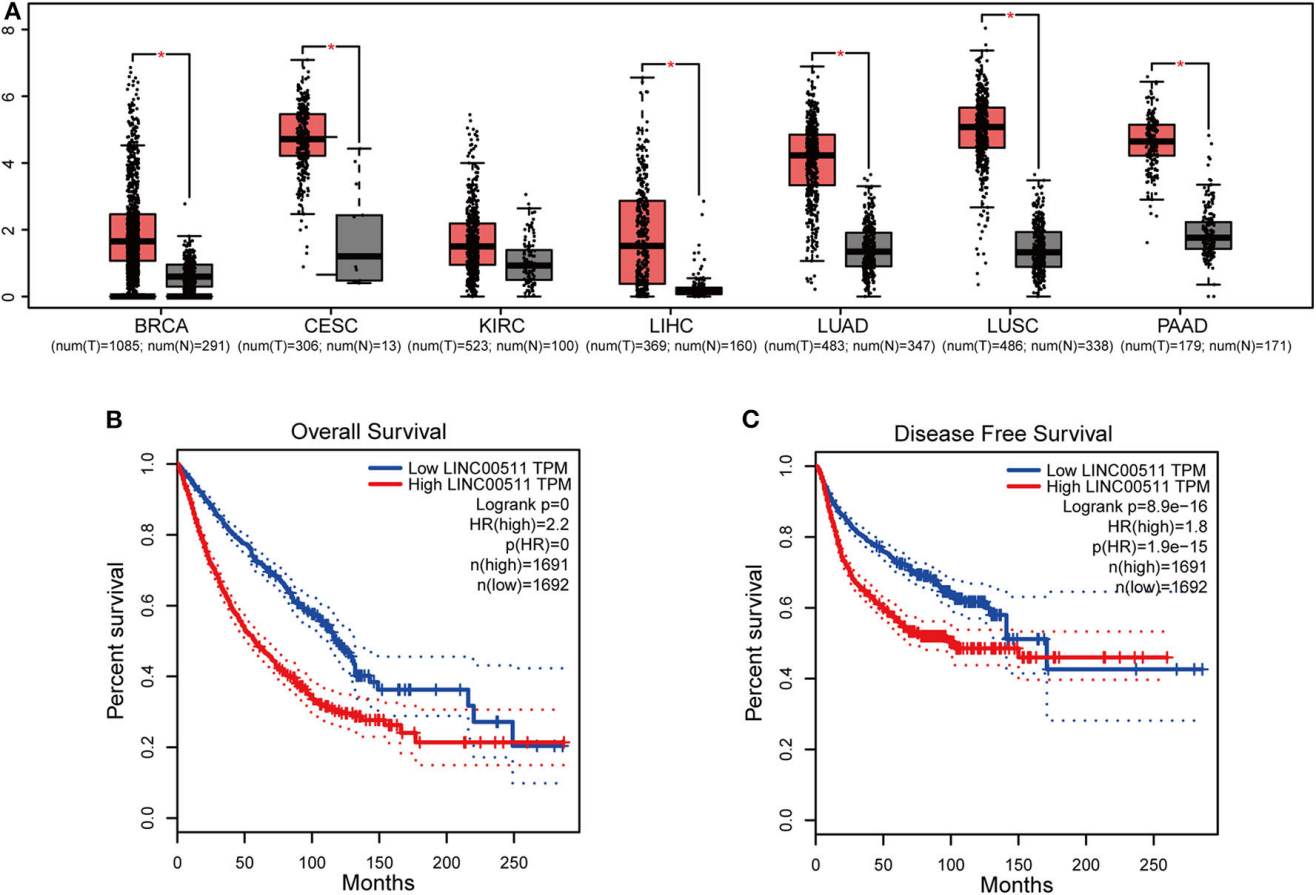
The expression level and prognostic significance of LINC00511 analyzed by cancer public database. **(A)** LINC00511 expression profile across tumor samples and adjacent normal tissues from GEPIA (*P < 0.01). **(B)** Overall survival and disease-free survival plots of LINC00511, LIHC, STAD, GBMLGG, BRCA, CESC, KIRC, LIHC, LUAD, LUSC, PAAD were included in (*n* = 3383, Log-rank *P* < 0.001). **(C)** Disease-free survival plots of LINC00511, LIHC, STAD, GBMLGG, BRCA, CESC, KIRC, LIHC, LUAD, LUSC, PAAD were included in (*n* = 3383, Log-rank *P* < 0.001).

We obtained LINC00511 expression across tumor samples and adjacent normal tissues and relevant prognostic information from GEPIA dataset to validate the results. LINC00511 was found over-expressed in most of the cancers, such as liver hepatocellular carcinoma, cervical squamous cell carcinoma and endocervical adenocarcinoma, lung adenocarcinoma, lung squamous cell carcinoma, breast invasive carcinoma, pancreatic adenocarcinoma (Figure 4A). Next, we combined LINC00511 with relevant prognostic information of cancers from digestive, respiratory and urinary system. According to the median expression of LINC0051, all 3,383 patients were classified to different LINC0051 expression group (high group or low group). Patients from low group had a favorable OS and DFS than those in high group (Log-rank *P* < 0.001, Figures 4B,C), as expected, confirming that LINC00511 is upregulated in various cancers and negatively correlated with OS and DFS.

### Function Analysis of LINC00511-Related Genes in Human Tumors

**Figure 5:**
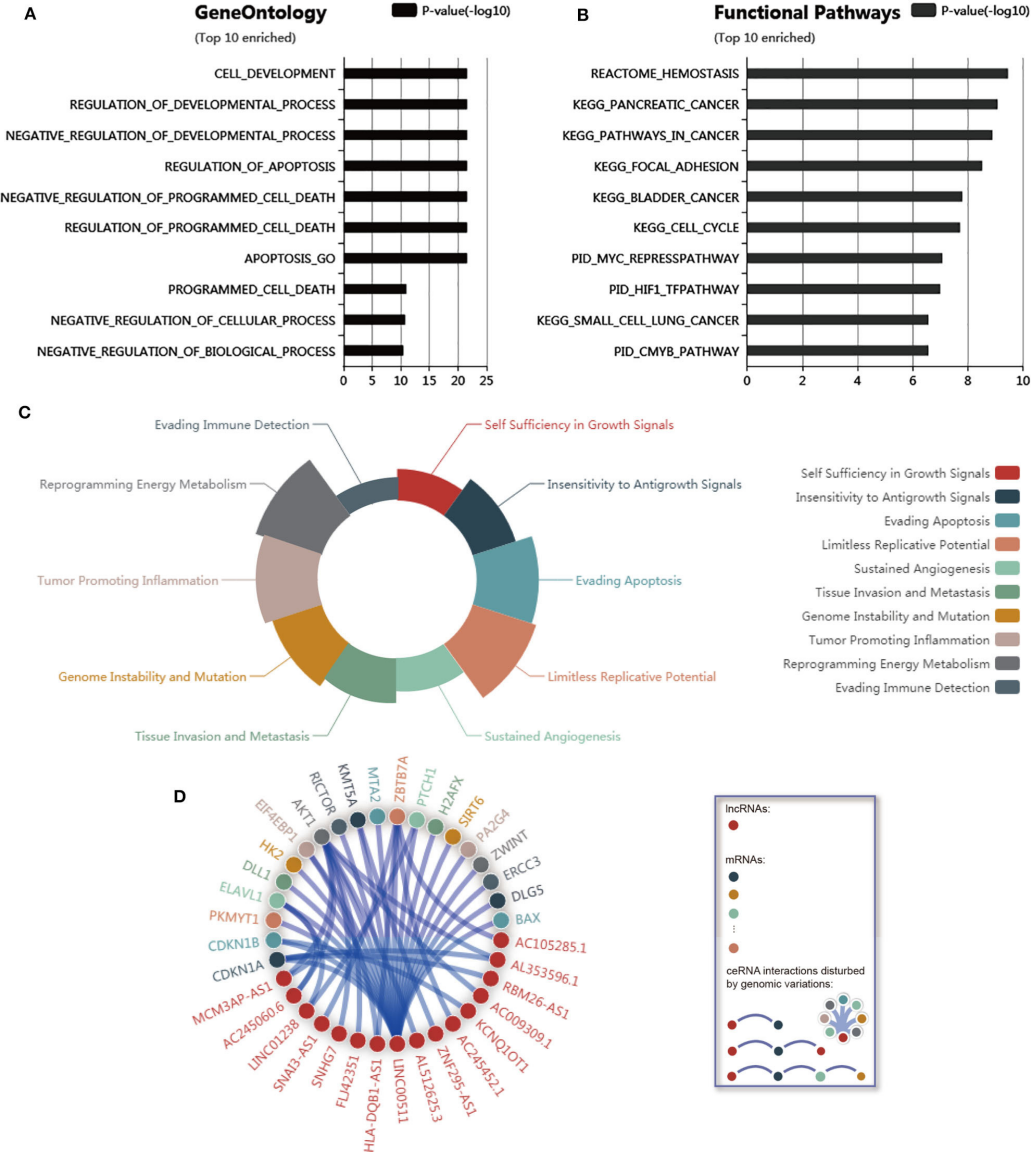
Correlation analysis of LINC00511. **(A)** Top 10 enriched gene ontology of LINC00511. **(B)** Top 10 enriched functional pathways of LINC00511. **(C)** Dysregulated hallmarks of variation-ceRNA event. **(D)** Related ceRNA network of LINC00511 disturbed by variation.

To further explore the function of LINC00511, genomic variations that disturb LINC00511-associated ceRNA network regulation was curated from LnCeVars dataset. Through gene ontology and functional pathways enrichment analysis, we found that gene ontology was mainly enriched in cell development and death, and apoptosis (Figure 5A), and functional pathways was mainly enriched in hemostasis, various cancers, focal adhesion, MYC, HIF1 and CMYB pathway (Figure 5B). Further, we found that dysregulated hallmarks of LINC00511-associated ceRNA event (Figure 5C). Related LINC00511-associated ceRNA network disturbed by genomic variations were about AKT1, ZBTB7A, CDKN1A, ELAVL1, MCM3AP-AS1, CDKN1B, PTCH1, KMT5A, AL353596.1, QB1-AS1, etc (Figure 5D). Finally, molecular mechanisms as well as functional characterizations of LINC00511 were summarized in Table 3.

**Table 3:** Functional characterizations and molecular mechanisms of LINC00511 in the included studies.

**Cancer type**	**Function**	**Interacted miRNA**	**Protein binding**	**Downstream molecules**
NSCLC	Cell proliferation, invasion, metastasis and apoptosis	-	EZH2	p57
BC	Cell proliferation, invasion, maintenance of cancer stem cells characteristic	miR-185-3p	-	E2F1, Nanog
PDA	Cell proliferation, invasion and tumor angiogenesis	miR-29b-3p	-	VEGFA
CcRCC	Cell proliferation, colony formation and metastasis	miR-625	-	CCND1
BC	Cell proliferation, apoptosis and radioresistance	miR-185	-	STXBP4
CC	Cell proliferation, invasion, metastasis and apoptosis	-	-	MRP1, P-GP, Bcl-2, MMP-2 and MMP-9
HC	Cell proliferation, invasion and metastasis	miR-424	-	-
CC	Cell proliferation, invasion and metastasis	-	-	-
BC	Cell proliferation and invasion	-	EZH2	CDKN1B

*BC, breast cancer; CC, cervical cancer; ccRCC, clear cell renal cell carcinoma; HC, hepatocellular carcinoma; NSCLC, non-small cell lung cancer; PDA, pancreatic ductal adenocarcinoma*.

## Discussion

LINC00511 shows cancer promoting activity because it was shown that LINC00511 is up-expressed in many cancers and related to several features of cancer, such as proliferation (Sun et al., 2016; Li et al., 2018; Lu et al., 2018; Deng et al., 2019; Liu et al., 2019; Mao et al., 2019; Yu et al., 2019; Zhang J. et al., 2019; Zhu et al., 2019), apoptosis (Sun et al., 2016; Li et al., 2018; Deng et al., 2019; Mao et al., 2019; Wang R. P. et al., 2019; Zhang J. et al., 2019; Zhu et al., 2019), invasion (Sun et al., 2016; Lu et al., 2018; Mao et al., 2019; Quan et al., 2019; Wang R. P. et al., 2019; Yu et al., 2019; Zhu et al., 2019), radio-resistance (Chen et al., 2019; Liu et al., 2019), migration (Sun et al., 2016; Mao et al., 2019; Wang R. P. et al., 2019; Yu et al., 2019; Zhu et al., 2019), drug resistance (Mao et al., 2019), metastasis (Deng et al., 2019; Zhu et al., 2019), and growth (Sun et al., 2016; Deng et al., 2019; Wang R. P. et al., 2019). High LINC00511 expression is significantly correlated with TNM classification, lymph node metastasis, and short OS among patients with clear cell renal cell carcinoma (Deng et al., 2019) and NSCLC (Zhu et al., 2019). Additionally, knockdown of LINC00511 restricted clear cell renal cell carcinoma cell proliferation, colony formation, and metastasis *in vitro*; accelerated cell cycle arrest at G0-G1 and apoptosis *in vitro*; and decreased tumor growth *in vivo* (Deng et al., 2019). This study presented the prognostic value of LINC00511 in various cancers. To our knowledge, our present study is the first meta-analysis about LINC00511. In our study, it is found that elevated LINC00511 expression had a statistically significant relationship with poor OS. From the cancer type subgroup analysis, LINC00511 can act as predictor of worse prognosis among various cancers, including breast cancer, digestive system cancer, and cervical cancer. The overall effect was similar in the stratified analysis according to HR obtain method, cut-off value, sample size and follow-up duration. Moreover, there was no publication bias. Although not all of the included studies discussed the relationship between LINC00511 and clinicopathological characteristics of cancers, we did a comprehensive analysis of the available data. We found that LINC00511 was related to age, clinical stage, tumor size and lymph node metastasis, but not gender (except breast cancer and cervical cancer), differentiation, and metastasis. When we carried out sensitivity analysis, the total HR after removing Sun et al. study (Sun et al., 2016) had a substantial change. We have reasons to believe that this study is the source of heterogeneity. In addition, meta-regression indicated follow-up time occupied most heterogeneity of the results.

Moreover, we further strengthened our results with survival information of other types of cancers retrieved from GEPIA. Results from GEPIA were highly consistent with those from meta-analysis. Then, we also conducted a functional analysis of LINC00511-related genes in human tumors to reveal possible mechanisms associated with poor prognosis. We found that gene ontology and LINC00511-associated ceRNA event mainly focuses on the development, death and apoptosis of cells, which is also consistent with the current research reports (Sun et al., 2016; Li et al., 2018; Lu et al., 2018; Deng et al., 2019; Liu et al., 2019; Mao et al., 2019; Wang R. P. et al., 2019; Yu et al., 2019; Zhang J. et al., 2019). Functional enrichment analysis in another study also revealed that LINC00511 was associated with focal adhesion in pancreatic cancer (Wang W. et al., 2019). There is also a relationship among focal adhesions, proliferation and MYC (Duperret et al., 2015). LINC00511 is involved in regulating immune system activation and its up-regulation is mostly resulted from gene instability (amplification) in the genome (Xu et al., 2017). LINC00511 can influence the prognosis of various tumors through different target genes or pathways (Quan et al., 2019; Zhang H. et al., 2019; Zhu et al., 2019), and the function is not exactly the same in different cancers.

Taken all together, the findings presented in this study give a suggestion that LINC00511-mediated diverse pathways might have implications with diverse tumors (Yan et al., 2019; Zhang H. et al., 2019). More and more evidences hold that lncRNAs can function as ceRNAs (Tay et al., 2014; Wang et al., 2019b). In particular, LINC00511 can sponge other microRNA, such as microRNA 29b-3p (Zhao et al., 2018; Quan et al., 2019), microRNA 29c (Zhang H. et al., 2019), microRNA 124-3p (Li et al., 2019), microRNA 185 (Lu et al., 2018; Liu et al., 2019), microRNA 195 (Hu et al., 2019), microRNA 424 (Wang R. P. et al., 2019), microRNA 618 (Guo et al., 2019), microRNA 625 (Deng et al., 2019), and microRNA 765 (Ding et al., 2018; Yan et al., 2019), to impact on cancer development, progression and prognosis. However, there is a rather limited number of studies available on major cancers, such as lung cancer, breast cancer, liver cancer, cervical cancer, brain cancer, and pancreatic cancer, and no relevant studies about esophageal cancer, thyroid cancer, stomach cancer, and colorectal cancer. Thus, more clinical trials on these cancers are needed.

Indeed, our study has some limitations. Firstly, we just included 9 studies after strict inclusion and exclusion criteria in the meta section, and some of them are small in sample size. Secondly, all the included studies were conducted in China, which is the reason why we did not conduct ethnic subgroup analysis to investigate any possible effect on the association between LINC00511 and clinical outcomes. Thirdly, some HRs was estimated rather than obtained directly. Finally, all included studies are retrospective, indicating a moderate risk of bias.

In summary, elevated LINC00511 is significantly related to age, clinical stage, tumor size, lymph node metastasis, and unfavorable clinical outcome in different types of cancers. Our results showed that LINC00511 may consider as a refreshing biomarker of lymph node metastasis and prognosis in patients with cancer. However, larger sample, multi-ethnic, prospective studies are warranted to confirm the prognostic value of LINC00511. Moreover, LINC00511 acts as ceRNA in the tumourigenesis and development, and this requires further study.

## Data Availability Statement

Publicly available datasets were analyzed in this study. This data can be found here: http://gepia.cancer-pku.cn/, http://www.bio-bigdata.net/LnCeVar/.

## Author Contributions

X-FW, BL, Y-HZ, and NG conceived, designed, or planned the study. BL and X-FW analyzed the data. CC and D-XZ acquired data. Y-XZ, NS, W-WN, WY, and J-AH helped interpret the results. X-FW and CC provided study materials or patients. D-XZ, BL, and Y-XZ drafted the manuscript. All authors revised and reviewed this work, and all authors gave their final approval of the submitted manuscript.

## Conflict of Interest

The authors declare that the research was conducted in the absence of any commercial or financial relationships that could be construed as a potential conflict of interest.

## Supplementary Material

Figure S1 Forest plots of hazard ratios for the association between LINC00511 expression and overall survival after excluding the study by Sun et al. (2016).

Figure S2 Forest plot of LINC00511 expression and OR for clinicopathological features. The investigated clinicopathological parameters are **(A)** gender, **(B)** age, **(C)** clinical stage, **(D)** tumor size, **(E)** differentiation, **(F)** LN metastasis and **(G)** distant metastasis.

Figure S3 Funnel plot for the meta-analyses of clinical.

Table S1 Detailed search strategies.

Table S2 The results of this meta-analysis and subgroup analyses for overall survival.
