# Supplementary figures and images for: Long Intergenic Non-protein Coding RNA 511 in Cancers
Source: Front Genet. 2020 Jul 7;11:667. doi: 10.3389/fgene.2020.00667 (PMC7358593; doi:10.3389/fgene.2020.00667)

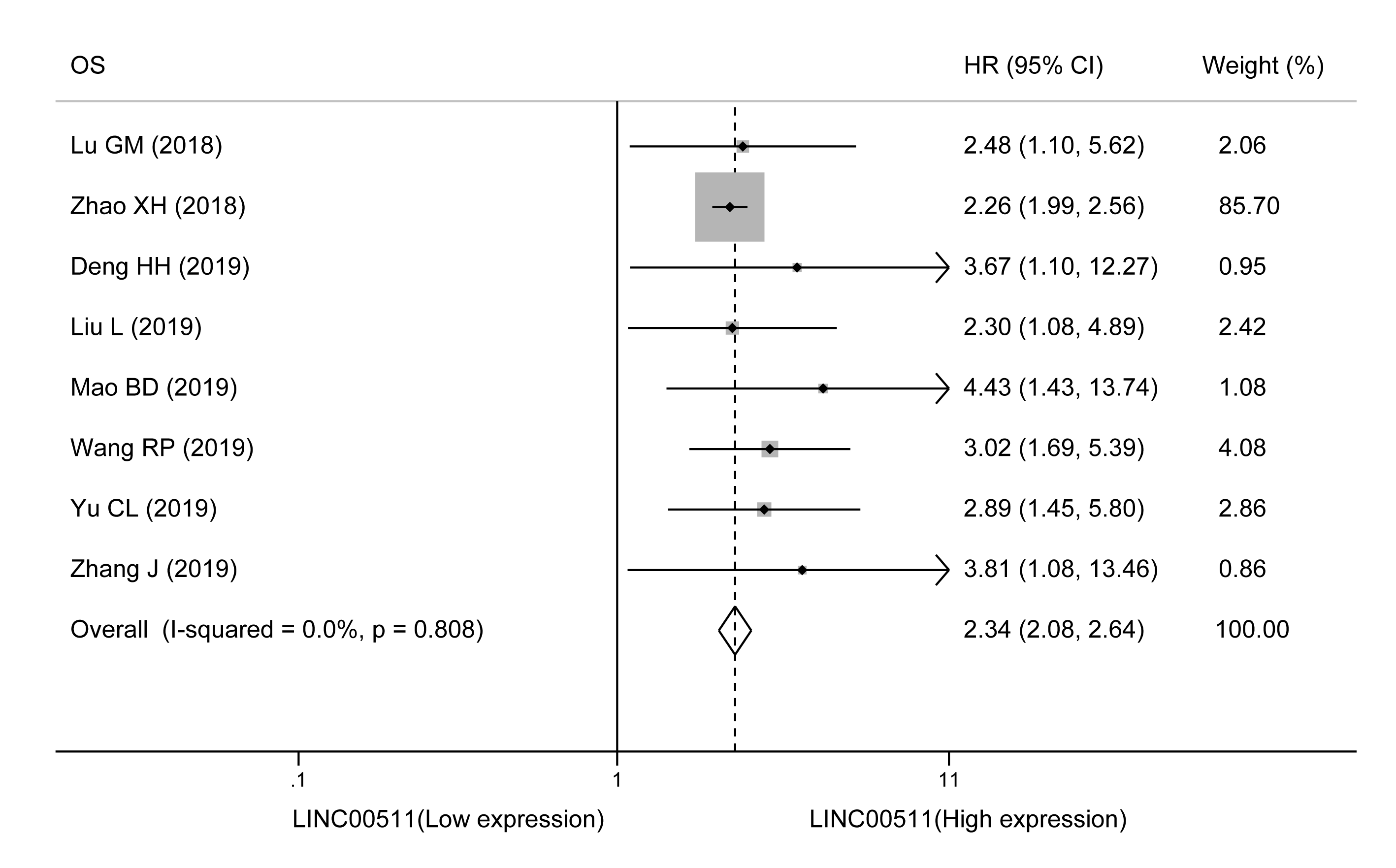

Supplement: Figure S1 — Forest plots of hazard ratios for the association between LINC00511 expression and overall survival after excluding the study by Sun et al. (2016). [file Image_1.TIF]

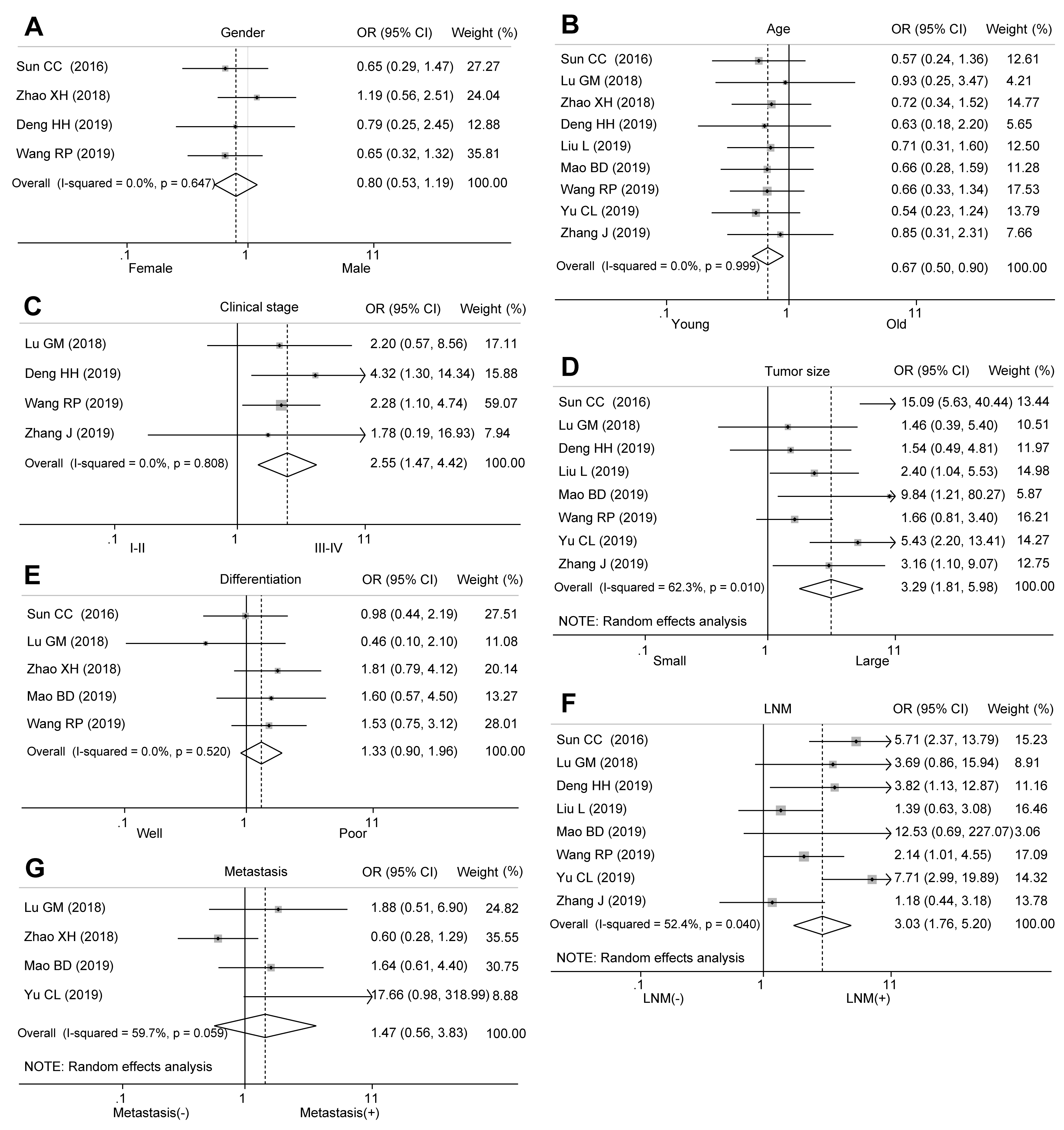

Supplement: Figure S2 — Forest plot of LINC00511 expression and OR for clinicopathological features. The investigated clinicopathological parameters are (A) gender, (B) age, (C) clinical stage, (D) tumor size, (E) differentiation, (F) LN metastasis and (G) distant metastasis. [file Image_2.TIF]

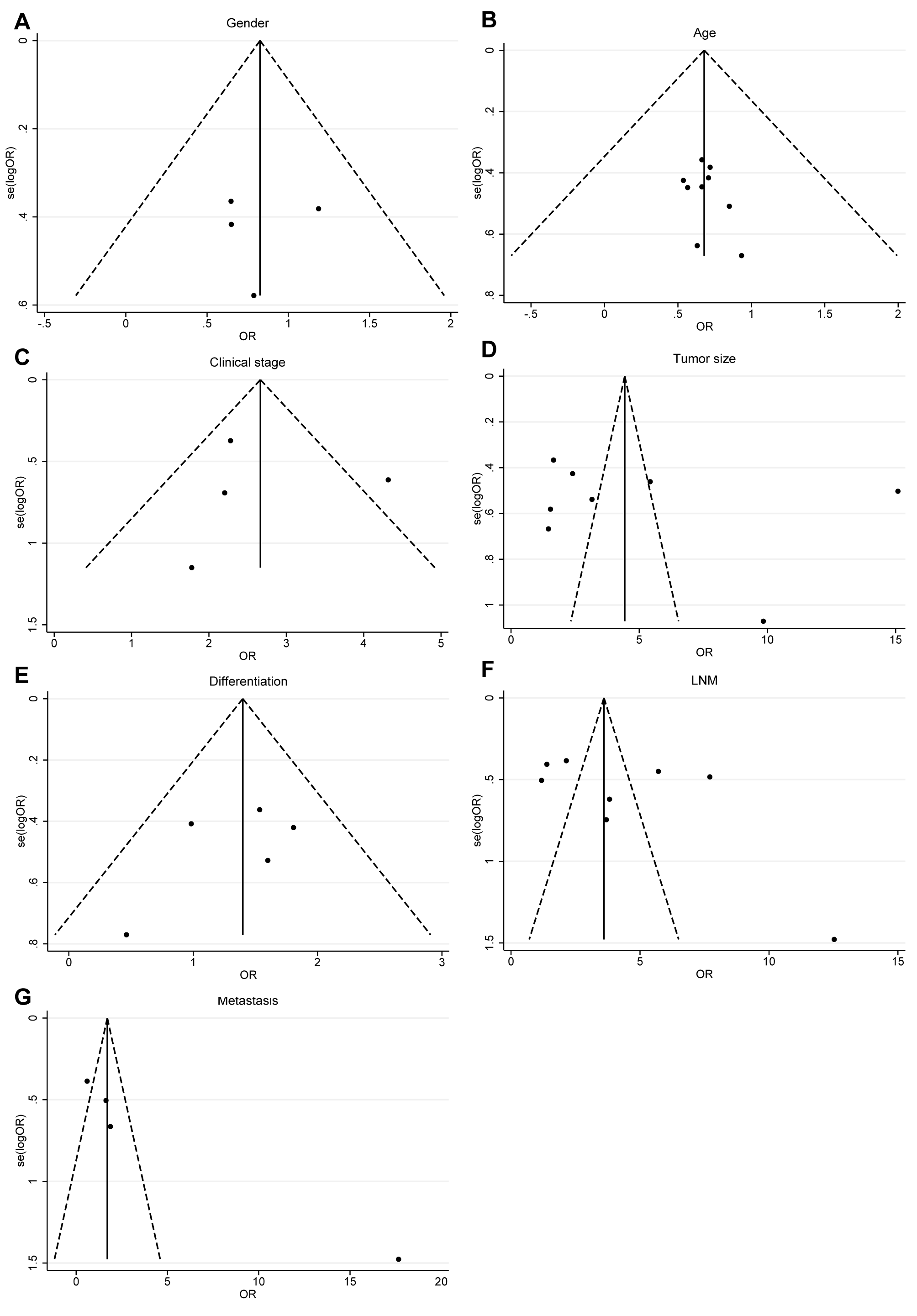

Supplement: Figure S3 — Funnel plot for the meta-analyses of clinical. [file Image_3.TIF]
