# Supplementary material for: Long Intergenic Non-protein Coding RNA 511 in Cancers
Source: Front Genet. 2020 Jul 7;11:667. doi: 10.3389/fgene.2020.00667 (PMC7358593; doi:10.3389/fgene.2020.00667)
Supplement: Table S1 — Detailed search strategies. [file Table_1.DOCX]

Table S1 Detailed search strategies.

| **Medline** | |
| --- | --- |
| #1 | “Neoplasms”[Mesh] |
| #2 | neoplasia[All fields] OR tumor[All fields] OR cancer[All fields] OR malignancy[All fields] OR carcinoma[All fields] |
| #3 | #1 OR #2 |
| #4 | LINC00511[All fields] OR Long Intergenic Non-Protein Coding RNA 511 [All fields] |
| #5 | #3 AND #4 Filters: Publication date to 2019/10/20 |
| **Embase** | |
| #1 | 'neoplasm'/exp |
| #2 | neoplasia OR tumor OR cancer OR malignancy OR carcinoma |
| #3 | #1 OR #2 |
| #4 | LINC00511[All fields] OR Long Intergenic Non-Protein Coding RNA 511 [All fields] |
| #5 | #3 AND #4 AND [<1966-2019]/py |
| **Web of Science** | |
| #1 | Neoplasms OR neoplasia OR tumor OR cancer OR malignancy OR carcinoma |
| #2 | LINC00511 OR Long Intergenic Non-Protein Coding RNA 511 |
| #3 | #1 AND #2 |
