# Supplementary material for: Long Intergenic Non-protein Coding RNA 511 in Cancers
Source: Front Genet. 2020 Jul 7;11:667. doi: 10.3389/fgene.2020.00667 (PMC7358593; doi:10.3389/fgene.2020.00667)
Supplement: Table S2 — The results of this meta-analysis and subgroup analyses for overall survival. [file Table_2.DOCX]

Table S2 The results of this meta-analysis and subgroup analyses for overall survival.

| Variables | Studies (n) | HR (95% CI) | *P*-value | I^2^ (%) | Model |
| --- | --- | --- | --- | --- | --- |
| **Overall survival** | 9 | 3.18 (2.29 ~ 4.42) | **< 0.001** | 58.10% | Random |
| **Cancer type** |  |  |  |  |  |
| BC | 3 | 2.57 (1.55 ~ 4.27) | **< 0.001** | 0.00% | Random |
| Digestive system | 2 | 2.29 (2.02 ~ 2.59) | **< 0.001** | 0.00% | Random |
| CC | 2 | 3.25 (1.80 ~ 5.88) | **< 0.001** | 0.00% | Random |
| Others | 2 | 6.41 (3.89 ~ 10.56) | **< 0.001** | 0.00% | Random |
| **HR obtain method** |  |  |  |  |  |
| OS R | 3 | 3.53 (1.67 ~ 7.45) | **0.001** | 87.90% | Random |
| OS Curve | 6 | 2.95 (2.08 ~ 4.17) | **< 0.001** | 0.00% | Random |
| **Cut-Off (high/low)** |  |  |  |  |  |
| Median | 5 | 2.97 (2.10 ~ 4.21) | **< 0.001** | 0.00% | Random |
| Others | 4 | 3.46 (1.76 ~ 6.81) | **< 0.001** | 82.20% | Random |
| **Sample sizes** |  |  |  |  |  |
| ≥ 100 | 3 | 3.54 (1.76 ~ 7.14) | **< 0.001** | 88.10% | Random |
| < 100 | 6 | 2.91 (2.01 ~ 4.20) | **< 0.001** | 0.00% | Random |
| **Follow-up time** |  |  |  |  |  |
| ≥ 60 months | 7 | 2.34 (2.08 ~ 2.63) | **< 0.001** | 0.00% | Random |
| < 60 months | 2 | 6.41 (3.89 ~ 10.56) | **< 0.001** | 0.00% | Random |

Note: Abbreviation: BC: breast cancer; CC: cervical cancer.
